# Real-World Effects of Melanopic-Enhanced Classroom Lighting on Sleep, Mood, and Cognition in Male Korean Adolescents: A Field-Based Pilot Study

**DOI:** 10.3390/clockssleep8010006

**Published:** 2026-01-30

**Authors:** Sumin Bae, Eunji Hwang, Ki-Young Jung

**Affiliations:** 1 Department of Neurology, Seoul National University Hospital, Seoul 03080, Republic of Korea; 2 Department of Medical Device, Seoul National University College of Medicine, Seoul 03080, Republic of Korea; 3 Neuroscience Research Institute, Seoul National University College of Medicine, Seoul 03080, Republic of Korea

**Keywords:** lighting, melanopic, students, adolescents, sleep, mood, cognition

## Abstract

Light exposure profoundly influences human emotions and physiology. Yet, adolescents spend considerable time under artificial indoor lighting. Reduced daytime light exposure delays the circadian clock, negatively affecting sleep, cognition, and mood. This pilot study examined whether 470–490 nm enhanced LED lighting modulates mood, sleep quality, and attention among 65 male Korean high school students (mean age = 15.4 years) who participated in a two-week intervention. Both groups were exposed to natural daylight, but the experimental group additionally used LED lighting enriched in the 470–490 nm wavelength range, whereas the control group used LED lighting without modified spectral characteristics. Students were exposed to the assigned lighting from 08:00 to 17:00 during regular school hours for two consecutive weeks. To evaluate the effects of the two-week intervention, pre- and post-assessments included the Beck Depression Inventory (BDI-II), the Richards–Campbell Sleep Questionnaire (RCSQ), the Epworth Sleepiness Scale (ESS), the Perceived Stress Scale (PSS), and the Frankfurter Attention Inventory (FAIR), administered twice at each assessment point. The linear mixed-effect model showed a significant time × group interaction for line errors in the first FAIR trial (F (1, 52) = 5.21, *p* = 0.027, η^2^ partial = 0.09), suggesting a greater relative reduction in attentional errors in the experimental group compared with the control group. No significant effects were observed for sleep- or mood-related outcomes. These results indicate the potential relevance of wavelength-optimized lighting in educational settings where sustained attention is critical. Future studies with larger samples and longer interventions are required to confirm and extend these findings.

## 1. Introduction

Light is a crucial factor influencing human physiological and psychological functions [1]. Short wavelengths, particularly at approximately 480 nm, are thought to play central roles in circadian rhythm regulation [2,3]. Short-wavelength light is detected by intrinsically photosensitive retinal ganglion cells (ipRGCs) containing melanopsin. These cells transmit signals to the suprachiasmatic nucleus (SCN) via the retinohypothalamic tract [4]. The stimulation of melanopsin by light quantified as melanopic illuminance provides a biologically relevant measure for assessing circadian effects [5,6]. Through this pathway, exposure to short-wavelength light in the morning is associated with circadian phase advance, while evening exposure has been linked to delayed melatonin onset [7]. Such shifts in circadian timing may consequently influence sleep [7], mood [8], cognitive function [9], alertness, and vigilance [10].

Adolescents live in environments that conflict with these biological characteristics. Although delayed sleep timing during adolescence—characterized by going to bed late and waking up late—is a normal developmental process [11], the school system forces them to study late into the night while still requiring early morning attendance. This places them in a state of chronic sleep deprivation [12]. The accumulation of sleep debt has been associated with adverse physical and mental health outcomes [13,14,15]. Furthermore, adolescents tend to spend most of their time in indoor spaces such as schools, private tutoring, and homes, significantly limiting their exposure to natural light while simultaneously living in a world saturated with artificial light [16,17]. In South Korea, adolescents face a particularly challenging light environment. Many spend extended hours in private tutoring institutes after school, confined to indoor settings throughout the day due to intense academic demands [18]. Such restricted exposure to daytime light, combined with extensive evening light exposure, may contribute to misaligned circadian timing, reduced sleep quality, and impairments in attention, potentially resulting in lower academic performance, as well as increased mental health issues such as depression and anxiety [19]. Despite growing recognition that optimizing the lighting environment may support student health and learning [20], empirical evidence examining these relationships under real-world educational conditions remains limited.

Exposure to blue-rich lighting in the morning has been suggested to be effective for improving sleep, mood, and cognitive function. A comparative lighting study involving university students analyzed the effects of one hour of exposure to blue-rich white light versus warm white light during the morning hours. While there were no group differences in physiological indicators such as cortisol, alertness and mood appeared to improve under blue-rich lighting [21]. Studies conducted in actual classroom environments with adolescents suggested even clearer cognitive effects related to learning. Cognitive processing speed and attention appeared to improve under blue-enhanced white lighting compared to conventional lighting [22]. Another study comparing exposure to red light and blue light in the morning and evening, respectively, suggested that morning blue light was most effective for enhancing attention [23]. A recent study indicated that strong morning light intensity in boarding schools had a significant effect on sleep quality and mood compared to strong evening light intensity [24]. However, despite these findings, research examining the effects of daytime classroom lighting—where adolescents spend the majority of their waking hours—remains limited. Moreover, the combined influence of lighting and other environmental conditions in real educational settings, rather than controlled laboratory environments, on students’ broader well-being, including sleep quality, cognitive performance, and mood, has not been comprehensively evaluated.

Therefore, this pilot study aims to examine the effects of 480 nm wavelength-enhanced lighting versus standard lighting on sleep quality, attention, and emotional changes in high school students within real classroom settings. Given the established sensitivity of the circadian system to short-wavelength light, it is reasonable to expect that enhanced 480 nm illumination may modulate multiple domains of daytime functioning. It is anticipated that a lighting environment rich in blue light will comprehensively improve high school students’ subjective sleep quality, attention, and mood. Consistent with this hypothesis, we found that melanopic-enhanced classroom lighting was associated with improvements in attentional performance, whereas no significant changes were observed in sleep quality or mood, highlighting the domain-specific effects of lighting in real-world educational settings.

## 2. Results

### 2.1. Lighting Conditions

Given that classrooms were located on different floor levels and positions within the building, we anticipated potential differences in illuminance. Illuminance was measured at nine predefined desk-level points in each classroom and averaged to obtain classroom-level values. The resulting mean illuminance levels were 1439 lx for Class 1, 1739 lx for Class 2, 2109 lx for Class 3, and 2250 lx for Class 4 (Table S1).

Three comparisons were conducted to assess illuminance differences by classroom location. First, the experimental group (Classes 3–4) exhibited a mean illuminance approximately 591 lx higher than that of the control group (Classes 1–2). Second, among first-floor classrooms located on opposite sides of the building, Class 3 showed a mean illuminance approximately 521 lx higher than that of the control group (Classes 1–2). Third, Class 4 (second floor) showed a mean illuminance approximately 141 lx higher than Class 3 (first floor).

CCT averaged 5092 K for Class 1, 5280 K for Class 2, 5246 K for Class 3, and 4923 K for Class 4. With the exception of Class 4, values remained within the target range of 5000–5300 K, and no significant differences in illuminance or CCT were observed between the control and experimental groups.

### 2.2. Intervention Effects on Sleep, Mood, and Attention

Sleep, mood, and attention outcomes were assessed at two time points (pre- and post-intervention) over a two-week period. Descriptive statistics of all outcome measures are summarized in Table 1.

In the first trial, line errors remained largely unchanged in the experimental group from before to after the intervention (0.3 ± 0.9 to 0.3 ± 1.0), whereas an increase was observed in the control group (0.3 ± 0.6 to 1.2 to 1.6) (Table 1). Consistent with these descriptive patterns, the linear mixed-effect model revealed a significant time × group interaction for line errors in the first trial (F (1, 52) = 5.21, *p* = 0.027, η^2^ partial = 0.09) (Table 2 and Table S2). Apart from this finding, no significant time × group interaction effects were significantly observed for other sleep or mood outcomes.

## 3. Discussion

In this study, we examined the effects of Q480 lighting on sleep, mood, and attention in high school students within a classroom environment. Participants were exposed to Q480 lighting—which enhances short-wavelength light (λmax: 470–490 nm) within the maximal spectral sensitivity range of melanopsin—during classes in their school life. We hypothesized that this short-wavelength-enhanced lighting would improve cognitive function, mood, and sleep. While no significant results emerged for sleep and mood, Q480 lighting significantly influenced one domain of attention assessment. Taken together, these findings suggest that wavelength-optimized lighting may exert its most immediate effects on attentional performance, whereas changes in subjective sleep quality and mood may require longer exposure durations or additional contributing factors to manifest.

Given the repeated-measures structure of FAIR, the presence of learning effects across sessions is expected [25,26]. Consistent with this expectation, significant main effects of time were observed for several performance indices. However, a statistically significant time × group interaction was detected only for line errors in the first trial, indicating a differential pattern of change between the experimental and control groups. Notably, this interaction reached statistical significance despite conservative inference arising from the limited degrees of freedom associated with classroom-level randomization, suggesting a signal that warrants further investigation in larger and more adequately powered studies. Line errors are not mere inaccuracies in response but rather attentional lapses that violate task rules—for instance, when a participant momentarily loses focus and marks an area outside the designated boundary instead of the correct target. Accordingly, a reduction in line errors indicates improvement in short-term concentration [22]. This specific improvement suggests that short-wavelength-enhanced lighting selectively enhanced sustained attentional accuracy rather than a broad enhancement of overall cognitive performance.

Most studies on blue-light exposure suggest that enhancing short-wavelength light during morning hours improves attentional performance [7,23,27]. However, the present study applied blue-light-rich lighting not just during the morning hours, but throughout the entire actual class period from 8 a.m. to 5 p.m., without any adjustments to students’ daily schedules. This approach aligns more closely with real educational lighting environments and suggests that cognitive abilities can improve not only with short-term, limited morning exposure but also with prolonged exposure throughout the entire daily learning period. Furthermore, while earlier studies often employed simulated classroom environments to verify attention enhancement [22], the present findings extend this evidence by demonstrating similar benefits under naturalistic classroom conditions where daylight exposure coexists with artificial lighting.

No statistically significant group differences were observed in perceived stress or depressive symptoms, likely due to substantial contextual variability in adolescent mood and the short two-week intervention period. Sleep quality and daytime sleepiness also did not change, as baseline levels were already close to the normal range, which may have contributed to a ceiling effect and reduced the sensitivity to detect further improvement. Overall, while melanopic-enhanced lighting may influence cognitive performance, longer interventions and populations with greater baseline impairment are needed to confirm these effects.

Adolescence is a period when most of the day is spent at school. Particularly in Korea, students mostly remain indoors in classrooms throughout the day and participate in private tutoring after school, resulting in minimal exposure to natural light [17]. Given this restricted access to daylight, one proposed strategy is to delay morning school start times, considering the characteristic delayed circadian rhythm observed during adolescence [28,29]. In fact, policies to delay school start times by one hour are being pursued in the United States and Europe from a public health perspective. Studies report that adjusting high school start times resulted in reduced tardiness and absenteeism, along with improved academic performance [30]. Additionally, increased sleep duration and reduced social jet lag were observed [31]. However, in Korea, adjusting school start times is not easily feasible due to institutional and societal constraints. Increasing daytime outdoor activities is another approach that could be considered, but this also presents implementation challenges due to differences in school curricula and environments. As an alternative, improving indoor lighting environments is gaining attention. The importance of light exposure in schools has been emphasized in numerous studies, with appropriately adjusted illuminance and color temperature reported to be closely related to students’ attention, alertness, and academic performance [31]. Consistent with this evidence, this study implemented lighting modifications within real classroom settings, demonstrating a significant improvement in attention. This result is consistent with reported enhancements in attentional performance and academic-related outcomes in prior studies [31]. Additionally, Sweden has implemented improvements to school lighting infrastructure, yielding positive evaluations and strong support from parents [31]. Thus, adjusting the lighting environment in educational spaces can be viewed as a practical and sustainable intervention strategy. Some prior studies suggest that while exposure to blue-enriched light during morning hours is effective, exposure during daytime hours has relatively limited effects [32], highlighting the importance of adjusting light intensity and spectral composition according to the time of day. Therefore, subsequent research should refine lighting parameters—including intensity, spectral composition, and timing of exposure—to establish more comprehensive and reproducible effects.

This study has several limitations. First, the wide confidence intervals observed for the interaction effects are likely attributable to the limited degrees of freedom for group-level estimates, resulting from the small number of randomized clusters. With only four classrooms serving as independent units, the precision of between-group and time x group interaction estimates was inherently constrained. As a result, effect size estimates should be interpreted cautiously. Second, the lighting intervention lasted only two weeks, a relatively short period, which limits the ability to assess longer-term effects. Third, the sample size may have constrained the generalizability of the findings. In addition, all participants were male students of the same age from a single school, further limiting the applicability of the results to broader adolescent population. Fourth, this study did not measure direct circadian markers such as chronotype, sleep timing, or circadian phase, which limits mechanistic interpretation of the observed effects. Moreover, the approximately 500 lx illuminance difference between groups represents a non-negligible variation that may have confounded the observed effects. Fifth, this study did not strictly control light-related variables in the natural classroom environment, nor did it control for smartphone use, after-school sleep, or other sources of light exposure, all of which may influence the results. Moreover, 24 h light exposure data were unavailable. Therefore, it remains unclear how much classroom lighting contributed relative to other light sources, limiting causal interpretation of the findings. Given the exploratory nature of this pilot investigation, future studies should address these limitations through longer periods, larger and more diverse samples, and stricter control of light exposure variables. However, it should be acknowledged that maintaining such stringent controls over extended periods in naturalistic settings remains challenging, which limits the ability to draw firm conclusions regarding long-term effects.

## 4. Materials and Methods

### 4.1. Participants Characteristics

This study was approved by the Institutional Review Board of Seoul National University Hospital (IRB No. 2409-150-1583; approval date: 3 February 2025). Participants were recruited from all four first-year classes. Of the 66 students invited, 65 healthy male adolescents (mean age: 15.4 ± 0.4 years) agreed to participate. No significant differences in age were observed between groups. Because participants were adolescents, written consent forms were obtained from all participants and their legal guardians. Consent forms were distributed to students in advance to allow them to review the document and discuss participation with their parents. Students submitted consent forms individually, ensuring anonymity so that peers or teachers could not identify each student’s participation decision. Inclusion criteria for participants were as follows: (1) no diagnosis of sleep or mood disorders; (2) no use of medications related to sleep or mental health. Exclusion criteria included blindness, significant visual impairment, or current treatment for sleep or mood disorders, and participants meeting any of these criteria were not enrolled in this study.

### 4.2. Field Study Setting and Participant Characteristics

This study was conducted during the spring of 2025 (late April to early May) at a high school located in Cheorwon, Gyeonggi Province, Republic of Korea (38.2107° N, 127.2165° E). Cheorwon is a rural area located in the northeastern part of South Korea. Weather conditions during the study period were predominantly clear [33]. The school building is a three-story structure with classrooms facing southeast, overlooking the playground. Given the rural surroundings and the absence of tall nearby structures, classrooms received largely unobstructed morning sunlight, helping to maintain relatively uniform ambient light conditions across days.

**Figure 1:**
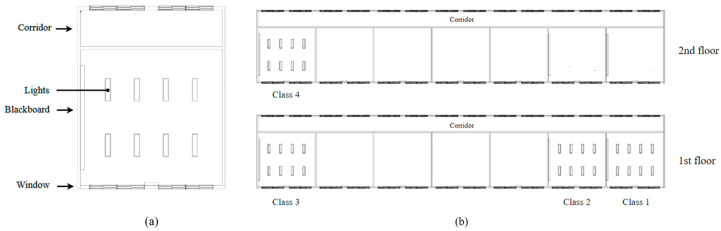
Classroom layout and classroom distribution. (**a**) Schematic diagram of a standard classroom layout illustrating the spatial arrangement of ceiling-mounted LED luminaires, blackboard, and windows. (**b**) Floor plan of the school building showing the distribution of the four first-year classrooms (Classes 1–4) across the first and second floors, connected by a central corridor.

The corridors are located on the north side of the building, and this layout provides good exposure to natural daylight. A total of four first-year classes participated in this study. Class 1 and Class 2 were located at the end of the first floor, Class 3 was on the opposite side of the first floor, and Class 4 was on the second floor (Figure 1). Random assignment was conducted at the classroom level, with two classes assigned to the experimental group and two to the control group.

To minimize bias, students were only informed that classroom lighting would change and that they would be asked to complete a questionnaire, with participants blinded to the specific allocation of experimental lighting. As a pilot, field-based study, students followed the school’s standard schedule. They attended classes from 08:00 to 17:00 Monday through Friday, according to the school timetable. Daily behaviors such as electronic device use, extracurricular activities, and bedtime routines were not controlled, allowing the intervention to be evaluated under naturalistic classroom conditions.

### 4.3. Lighting Installation

**Figure 2:**
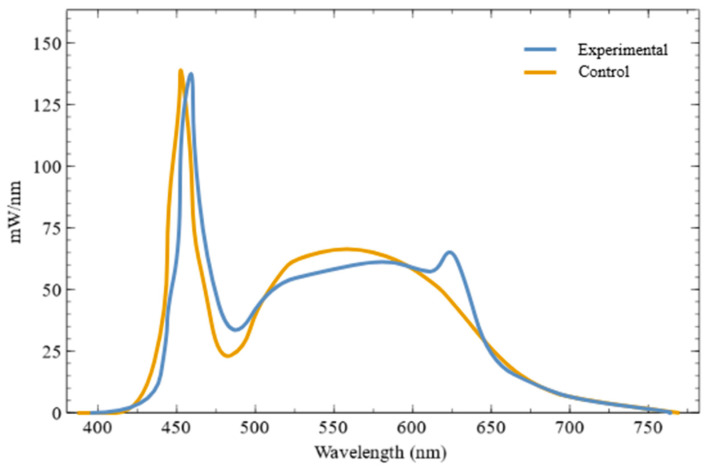
Spectral power distribution of the experimental LED and the control LED, showing enhanced short-wavelength content in the experimental LED compared to the control LED.

The experimental classrooms were equipped with ceiling-mounted Q480 LED lighting fixtures (BY THE M, Incheon, Republic of Korea). The lighting fixture (BME-4057-31QS) used in the experimental conditions was designed to enhance the melanopic effects while reducing potential blue light hazard compared to conventional fluorescent lamps or the control LED system (BME-4057-31NN). Specifically, both types had the same correlated color temperature (CCT) (5700 K), but the experimental LED exhibited approximately 5% lower spectral power in the blue light hazard range (420–460 nm) and approximately 30% higher spectral power in the melanopic range (470–490 nm) [34]. The color rendering index (Ra) was slightly lower for the experimental luminaire (83.9) than for the control luminaire (90.0) (Table 3 and Figure 2). Lighting intervention was conducted in two designated experimental classrooms, where existing fluorescent fixtures were replaced with the Q480 LED system. Two control classrooms were equipped with lighting fixtures identical in form and size to those used in the experimental group, but with no alterations in spectral characteristics, serving as standard illumination conditions. A total of eight lighting fixtures arranged in a 2 × 4 configuration were installed in each classroom (Figure 1). Classroom layouts were consistent across all rooms, with a blackboard positioned on the west wall, windows facing the playground on the south, hallway-facing windows on the north, and a solid wall on the east, ensuring comparable spatial and lighting geometry between experimental and control classrooms.

Illuminance (lx) and CCT (K) were measured under clear-sky conditions between 12:00 p.m. and 1:00 p.m. before and after lighting installation. All measurements were conducted at desk height (0.75 m above floor level) using optical measurement equipment (BY THE M) to ensure standardized evaluation of the lighting environment. Daylight in the classroom environment was not experimentally controlled. All participating classrooms were southeast-facing with unobstructed window views, allowing for direct solar exposure during the measurement period. Window blind usage was not controlled and was left to the discretion of teachers and students.

### 4.4. Design and Procedure

This study was designed as a cluster-randomized controlled pilot trial. Participants comprised all four first-year classes at one high school. Through random assignment, two classes were assigned to the experimental group (Q480 LED lighting), while the remaining two classes were assigned to the control group.

This study was conducted over a three-week period, beginning with a one-week baseline phase, followed by a two-week lighting intervention, and concluding with post-intervention assessments. Subjective measures of sleep, mood, and attention were administered at both baseline and post-intervention, allowing changes in these outcomes to be evaluated within and between groups.

### 4.5. Questionnaires and Attention Test

Four standardized psychometric instruments assessing sleep quality, daytime sleepiness, depressive symptoms, and perceived stress, along with the Frankfurt Attention Inventory (FAIR) for attention abilities, were used to evaluate key domains of sleep, mood, and attention. These measures provided a comprehensive set of subjective indices, allowing multiple aspects of students’ daily functioning to be assessed.

The Richards–Campbell Sleep Questionnaire (RCSQ) is a 5-item self-report scale that assesses subjective sleep quality using a 0–100 point visual analog rating. Each item evaluates a different aspect of sleep (e.g., depth, latency, and nighttime awakenings), with higher scores reflecting better sleep quality [35]. The Epworth Sleepiness Scale (ESS) is an 8-item questionnaire designed to quantify daytime sleepiness in everyday situations. Each item is rated on a 4-point Likert scale (0–3), yielding a total score ranging from 0 to 24, where a higher score indicates more severe daytime sleepiness [36].

The Beck Depression Inventory (BDI) is a 21-item self-report scale measuring the severity of depressive symptoms. Each item is rated on a 4-point Likert scale (0–3), yielding a total score ranging from 0 to 63, with higher scores indicating more severe depressive symptoms [37]. The Perceived Stress Scale (PSS) is a 10-item scale measuring the degree to which an individual evaluates life situations as stressful. Items are rated on a 5-point Likert scale (0–4), with higher scores indicating greater perceived stress [38].

The Frankfurt Attention Test (FAIR) was used to quantitatively assess attention and cognitive abilities [39,40]. This test consists of two parallel forms, each containing 320 items, administered under time constraints. Scoring yields a total performance score (T) and three indices derived from correct and error analysis: performance (P), quality (Q), and consistency (C). Error types include line errors (E(L)), omission errors (E(O)), and commission errors (E(C)).

### 4.6. Statistical Analysis

All statistical analyses were performed using R software (R, version 4.5.0). Outcome measures included sleep-related questionnaires (RCSQ and ESS), mood assessment tools (BDI and PSS), and the Frankfurt Attention Inventory (FAIR) for attention assessment. Only participants who completed both pre- and post-intervention assessments were included in the analysis, and participants with incomplete questionnaire data were excluded from the respective analyses.

Baseline characteristics were summarized using descriptive statistics. The primary analysis of intervention effects was performed using linear mixed-effect models with fixed effects for group, time (pre vs. post), and their interaction (group × time), with random intercepts specified for participants and classroom.

F statistics are reported with numerator degrees of freedom of 1. Denominator degrees of freedom for group effects were 2 due to classroom-level randomization (four clusters). Denominator degrees of freedom for time and group × time effects were estimated using the Satterthwaite approximation. Effect sizes are reported as partial eta-squared (η^2^ partial) and 95% confidence intervals.

## 5. Conclusions

The preliminary results of the present study suggested that Q480 lighting with enhanced melanopic wavelengths may contribute to selective changes in attentional performance in real classroom environments. These findings are consistent with previous research and provide further empirical evidence for the potential benefits of human-centric lighting in educational settings. Future studies with larger and more diverse samples, as well as extended intervention durations, are warranted to evaluate and clarify these findings.

## Figures and Tables

**Table 1:** Descriptive statistics, including the mean and standard deviation of all measurements.

	Experiment		Control	
	Before	After	Before	After
**Sleep**				
ESS	6.4 (4.0)	6.3 (4.1)	8.1 (3.4)	8.5 (3.8)
RCSQ	408.6 (71.4)	408.6 (79.7)	368.1 (66.8)	384.4 (96.8)
RCSQ 1	1 79.7 (19.4)	82.9 (19.2)	73.6 (21.3)	75.9 (25.6)
RCSQ 2	74.8 (19.0)	82.9 (16.5)	72.1 (20.1)	75.6 (21.5)
RCSQ 3	84.8 (20.6)	83.9 (20.2)	79.3 (20.2)	78.9 (27.9)
RCSQ 4	89.3 (21.5)	85.9 (20.6)	72.1 (32.8)	81.5 (24.6)
RCSQ 5	77.9 (20.6)	79.6 (20.6)	74.3 (19.5)	74.4 (24.9)
**Mood**				
PSS	15.7 (5.1)	16.2 (5.6)	18.4 (3.9)	17.3 (4.9)
BDI	10.9 (7.7)	9.0 (7.5)	12.5 (7.7)	11.3 (7.9)
**Attention**				
P	374.8 (95.1)	503.4 (113.8)	393.2 (111.0)	515.8 (107.7)
Q	1.0 (0.0)	1.0 (0.1)	0.9 (0.1)	1.0 (0.1)
C	367.6 (92.3)	483.9 (126.0)	375.4 (117.3)	494.3 (115.9)
**1st Trial**				
T	183.9 (44.8)	256.4 (49.5)	183.7 (53.1)	259.4 (60.0)
E(L)	0.3 (0.9)	0.3 (1.0)	0.3 (0.6)	1.2 (1.6)
E(O)	1.3 (2.3)	3.7 (9.1)	3.1 (4.4)	3.1 (5.2)
E(C)	0.8 (1.5)	1.8 (1.9)	2.1 (4.2)	2.0 (2.6)
**2nd Trial**				
T	217.4 (48.7)	270.9 (57.0)	233.7 (48.8)	280.1 (44.1)
E(L)	0.9 (2.2)	1.7 (4.1)	0.9 (2.1)	2.1 (2.7)
E(O)	2.1 (2.4)	3.8 (7.3)	4.1 (7.8)	3.6 (4.0)
E(C)	0.8 (1.5)	1.7 (2.0)	2.2 (4.9)	1.4 (2.2)

Values are presented as mean (standard deviation). Abbreviation: ESS, Epworth Sleepiness Scale; RCSQ, Richards–Campbell Sleep Questionnaire; PSS, Perceived Stress Scale; BDI, Beck Depression Inventory; P, performance; Q, quality; C, consistency; T, total item; E(L), line error; E(O), omission error; E(C), commission error.

**Table 2:** Results of the linear mixed-effect models.

	Time			Group			Time × Group		
	F	*p*	η^2^ Partial	F	*p*	η^2^ Partial	F	*p*	η^2^ Partial
**Sleep**									
ESS	0.18	0.671	0.00	3.01	0.225	0.60	0.33	0.566	0.00
RCSQ	0.52	0.475	0.01	2.09	0.286	0.51	0.11	0.745	0.00
RCSQ1	0.89	0.349	0.02	1.25	0.380	0.38	0.01	0.921	0.00
RCSQ2	3.73	0.059	0.07	1.14	0.397	0.36	0.62	0.433	0.01
RCSQ3	0.11	0.747	0.00	0.92	0.438	0.32	0.04	0.848	0.00
RCSQ4	0.02	0.881	0.00	0.40	0.590	0.17	1.44	0.236	0.03
RCSQ5	0.06	0.808	0.00	0.51	0.549	0.20	0.01	0.923	0.00
**Mood**									
PSS	0.33	0.565	0.00	2.95	0.228	0.60	2.17	0.146	0.04
BDI	8.39	0.005	0.13	1.04	0.415	0.34	0.41	0.526	0.00
**Attention**									
P	159.89	<0.001	0.74	0.82	0.461	0.29	0.80	0.374	0.01
Q	0.01	0.912	0.00	3.40	0.207	0.63	0.00	0.958	0.00
C	143.85	<0.001	0.72	0.35	0.612	0.15	0.42	0.522	0.00
**1st trial**									
T	213.59	<0.001	0.80	0.14	0.747	0.06	0.00	0.951	0.00
E(L)	6.47	0.014	0.11	4.40	0.171	0.69	5.21	**0.027**	0.09
E(O)	0.27	0.603	0.00	1.95	0.298	0.49	1.06	0.308	0.02
E(C)	2.93	0.093	0.05	0.80	0.466	0.29	0.90	0.347	0.02
**2nd trial**									
T	105.02	<0.001	0.67	0.75	0.478	0.27	0.30	0.584	0.00
E(L)	3.10	0.084	0.06	0.88	0.448	0.30	2.03	0.16	0.04
E(O)	0.07	0.787	0.00	1.45	0.352	0.42	0.25	0.622	0.00
E(C)	0.26	0.613	0.00	0.04	0.868	0.02	1.24	0.272	0.02

Statistical analyses were performed using linear mixed-effect models. Detailed degrees of freedom and 95% confidence intervals are reported in Supplementary Table S2. Abbreviation: ESS, Epworth Sleepiness Scale; RCSQ, Richards–Campbell Sleep Questionnaire; PSS, Perceived Stress Scale; BDI, Beck Depression Inventory; P, performance; Q, quality; C, consistency; T, total item; E(L), line error; E(O), omission error; E(C), commission error.

**Table 3:** Photometric and spectral measurements of Q480 LED versus conventional LED.

	Control	Experiment
Product model	BME-4057-31NN	BME-4057-31QS
Rated power (W)	40	40
Correlated color temperature (K)	5700	5700
Blue-light hazard wavelength SPD (420–460 nm, mW)	2688.4	2283.8
Melanopic wavelength SPD (470–490 nm, mW)	584.7	850.8
Color rendering index (Ra)	83.9	90.0

Abbreviations: LED, light-emitting diode; SPD, spectral power distribution; Ra, color rendering index.

## Data Availability

The data that support the findings of this study are available from the corresponding author upon reasonable request. Due to privacy and ethical restrictions, the data are not publicly available.

## Supplementary Materials

The following supporting information can be downloaded at https://www.mdpi.com/article/10.3390/clockssleep8010006/s1.

## Abbreviations

The following abbreviations are used in this manuscript:

BDI	Beck Depression Inventory
C	Consistency
CCT	Correlated color temperature
ESS	Epworth Sleepiness Scale
E(C)	Commission error
E(L)	Line error
E(O)	Omission error
LED	Light-emitting diode
P	Performance
PSS	Perceived Stress Scale
Q	Quality
Ra	Color rendering index
RCSQ	Richards–Campbell Sleep Questionnaire
SPD	Spectral power distribution
T	Total item
