# Supplementary material for: Real-World Effects of Melanopic-Enhanced Classroom Lighting on Sleep, Mood, and Cognition in Male Korean Adolescents: A Field-Based Pilot Study
Source: Clocks Sleep. 2026 Jan 30;8(1):6. doi: 10.3390/clockssleep8010006 (PMC12922084; doi:10.3390/clockssleep8010006)
Supplement: Supplementary file 1 [file clockssleep-08-00006-s001.zip › clockssleep-4037898-supplementary.pdf]

Supplementary Table S1. Classroom Lighting Conditions.

	**Control**		**Experimental**		***p***
	Class 1	Class 2	Class 3	Class 4	
Illuminance (lx)	1439	1739	2109	2250	0.070*
Correlated color temperature (K)	5092	5280	5246	4923	0.640*

Values are presented as means. Between-group differences were analyzed using independent t-tests.

* (asterisk) indicate comparisons between the control group (Classes 1–2) and the experimental group (Classes 3–4)

Supplementary Table S2. Results of linear mixed-effects models for sleep, mood, and attention outcomes

		F(df)	p	η^2partial	95% CI
**sleep**					
ESS	Time	0.18 (1,57)	0.671	0.00	[0.00, 1.00]
	Group	3.01 (1,2)	0.225	0.6	[0.00, 1.00]
	Time × Group	0.33 (1,57)	0.566	0.00	[0.00, 1.00]
RCSQ	Time	0.52 (1,49)	0.475	0.01	[0.00, 1.00]
	Group	2.09 (1,2)	0.286	0.51	[0.00, 1.00]
	Time × Group	0.11 (1,49)	0.745	0.00	[0.00, 1.00]
RCSQ1	Time	0.89 (1,53)	0.349	0.02	[0.00, 1.00]
	Group	1.25 (1,2)	0.38	0.38	[0.00, 1.00]
	Time × Group	0.01 (1,53)	0.921	0.00	[0.00, 1.00]
RCSQ2	Time	3.73 (1,53)	0.059	0.07	[0.00, 1.00]
	Group	1.14 (1,2)	0.397	0.36	[0.00, 1.00]
	Time × Group	0.62 (1,53)	0.433	0.01	[0.00, 1.00]
RCSQ3	Time	0.11 (1,53)	0.747	0.00	[0.00, 1.00]
	Group	0.92 (1,2)	0.438	0.32	[0.00, 1.00]
	Time × Group	0.04 (1,53)	0.848	0.00	[0.00, 1.00]
RCSQ4	Time	0.02 (1,49)	0.881	0.00	[0.00, 1.00]
	Group	0.40 (1,2)	0.59	0.17	[0.00, 1.00]
	Time × Group	1.44 (1,49)	0.236	0.03	[0.00, 1.00]
RCSQ5	Time	0.06 (1,53)	0.808	0.00	[0.00, 1.00]
	Group	0.51 (1,2)	0.549	0.2	[0.00, 1.00]
	Time × Group	0.01 (1,53)	0.923	0.00	[0.00, 1.00]
**mood**					
BDI	Time	8.39 (1,57)	0.005	0.13	[0.02, 1.00]
	Group	1.04 (1,2)	0.415	0.34	[0.00, 1.00]
	Time × Group	0.41 (1,57)	0.526	0.00	[0.00, 1.00]
PSS	Time	0.33 (1,58)	0.565	0.00	[0.00, 1.00]
	Group	2.95 (1,2)	0.228	0.6	[0.00, 1.00]
	Time × Group	2.17 (1,58)	0.146	0.04	[0.00, 1.00]
**Attention**					
P	Time	159.89 (1,56)	<0.001	0.74	[0.64, 1.00]
	Group	0.82 (1,2)	0.461	0.29	[0.00, 1.00]
	Time × Group	0.80 (1,56)	0.374	0.01	[0.00, 1.00]
Q	Time	0.01 (1,55)	0.912	0.00	[0.00, 1.00]
	Group	3.40 (1,2)	0.207	0.63	[0.00, 1.00]
	Time × Group	0.00 (1,55)	0.958	0.00	[0.00, 1.00]
C	Time	143.85 (1,56)	<0.001	0.72	[0.62, 1.00]

	Group	0.35 (1,2)	0.612	0.15	[0.00, 1.00]
	Time × Group	0.42 (1,56)	0.522	0.00	[0.00, 1.00]
first trial					
T	Time	213.59 (1,52)	<0.001	0.8	[0.72, 1.00]
	Group	0.14 (1,2)	0.747	0.06	[0.00, 1.00]
	Time × Group	0.00 (1,52)	0.951	0.00	[0.00, 1.00]
E(L)	Time	6.47 (1,52)	0.014	0.11	[0.01, 1.00]
	Group	4.40 (1,2)	0.171	0.69	[0.00, 1.00]
	Time × Group	5.21 (1,52)	0.027	**0.09**	[0.01, 1.00]
E(O)	Time	0.27 (1,52)	0.603	0.00	[0.00, 1.00]
	Group	1.95 (1,2)	0.298	0.49	[0.00, 1.00]
	Time × Group	1.06 (1,52)	0.308	0.02	[0.00, 1.00]
E(C)	Time	2.93 (1,52)	0.093	0.05	[0.00, 1.00]
	Group	0.80 (1,2)	0.466	0.29	[0.00, 1.00]
	Time × Group	0.90 (1,52)	0.347	0.02	[0.00, 1.00]
second trial					
T	Time	105.02 (1,52)	<0.001	0.67	[0.64, 1.00]
	Group	0.75 (1,2)	0.478	0.27	[0.00, 1.00]
	Time × Group	0.30 (1,52)	0.584	0.00	[0.00, 1.00]
E(L)	Time	3.10 (1,52)	0.084	0.06	[0.00, 1.00]
	Group	0.88 (1,2)	0.448	0.3	[0.00, 1.00]
	Time × Group	2.03 (1,52)	0.16	0.04	[0.00, 1.00]
E(O)	Time	0.07 (1,52)	0.787	0.00	[0.00, 1.00]
	Group	1.45 (1,2)	0.352	0.42	[0.00, 1.00]
	Time × Group	0.25 (1,52)	0.622	0.00	[0.00, 1.00]
E(C)	Time	0.26 (1,52)	0.613	0.00	[0.00, 1.00]
	Group	0.04 (1,2)	0.868	0.02	[0.00, 1.00]
	Time × Group	1.24 (1,52)	0.272	0.02	[0.00, 1.00]

Linear mixed-effects models were fitted with fixed effects for Group, Measurement Point (pre vs. post), and their interaction (Time × Group), and random intercepts for participants and classrooms. F statistics are reported with numerator degrees of freedom of 1. Denominator degrees of freedom for group effects were 2 due to classroom-level randomization (four clusters). Denominator degrees of freedom for time and time × group effects were estimated using the Satterthwaite approximation. The intervention effect was evaluated based on the time × group interaction. Partial eta-squared (η^2 partial) and 95% confidence intervals are reported
